# Influence of Preparation Procedure on Physicochemical and Antibacterial Properties of Titanate Nanotubes Modified with Silver

**DOI:** 10.3390/nano9050795

**Published:** 2019-05-23

**Authors:** Manu Jose, Paulina Sienkiewicz, Karolina Szymańska, Dominika Darowna, Dariusz Moszyński, Zofia Lendzion-Bieluń, Kacper Szymański, Sylwia Mozia

**Affiliations:** 1 Institute of Inorganic Chemical Technology and Environment Engineering, Faculty of Chemical Technology and Engineering, West Pomeranian University of Technology, Szczecin, ul. Pułaskiego 10, 70-322 Szczecin, Poland; manu.jose@zut.edu.pl (M.J.); paulina.sienkiewicz@zut.edu.pl (P.S.); dominika.darowna@zut.edu.pl (D.D.); dariusz.moszynski@zut.edu.pl (D.M.); zofia.lendzion-bielun@zut.edu.pl (Z.L.-B.); kacper.szymanski@zut.edu.pl (K.S.); 2 Nanomaterials Physicochemistry Department, Faculty of Chemical Technology and Engineering, West Pomeranian University of Technology, Szczecin, al. Piastów 45, 70-311 Szczecin, Poland; karolina.szymanska@zut.edu.pl

**Keywords:** titanate nanotubes, TNT, Ag, antibacterial, *Escherichia coli*, *Staphylococcus epidermidis*

## Abstract

Silver nanoparticles (NPs) are effective antibacterial agents; however, aggregation of NPs and uncontrolled release of Ag^+^ affect their efficiency and may pose a risk to the environment. To overcome these disadvantages, immobilization of Ag onto titanate nanotubes (TNTs) was investigated. This paper describes the physicochemical and antibacterial properties of silver incorporated titanate nanotubes (Ag/TNTs) prepared using five procedures and containing different Ag amounts (0.11–30.85 wt.%). The methods were (i) sol-gel followed by a hydrothermal process; (ii) photodeposition under ambient conditions; (iii) photodeposition under an inert atmosphere; (iv) NaBH_4_ reduction; and (v) electroless deposition after activation of TNTs with Sn^2+^. Depending on the synthesis procedure, the presence of metallic Ag NPs, AgO or AgCl was observed. The electroless method led to an additional deposition of SnO_2_ NPs. The antibacterial properties of Ag/TNTs were analyzed as a function of Ag content and released against *Escherichia coli* and *Staphylococcus epidermidis*. The best bactericidal properties exhibited Ag/TNTs prepared through the photodeposition process due to the higher interaction of exposed Ag NPs with bacteria. An increase of Ag loading resulted in improvement of antibacterial activity of Ag/TNTs although no direct correlation between silver content or release and inhibition of bacterial growth was found.

## 1. Introduction

The presence of large concentrations of microorganisms such as bacteria, protozoans, and viruses in the majority of the world’s water resources limits the use of a major portion of them as drinking water. According to the World Health Organization (WHO), 80% of diseases arise due to the consumption of contaminated water [1]. Hence, the development of new efficient technologies for the removal of harmful microorganisms from water will require much attention in the coming decades. Recently, nanomaterials with superior photoactivity, high surface-to-volume ratios, antibacterial properties, and good hydrophilicity have been explored for the inactivation of pathogenic microorganisms. Among them, silver NPs have gained much attention for their powerful antibacterial properties [2,3] which find application in wound dressing [4], textiles [5], and self-sterilizing surfaces in food and pharmaceutical production [6]. The Ag NPs show good antimicrobial properties towards a broad spectrum of bacterial and fungal species including antibiotic-restraint strains [7,8].

Even though Ag NPs are known as promising biocidal agents due their distinctive physicochemical properties, their wide applicability is limited mainly due to the uncontrolled release of Ag^+^ ions from the Ag NPs, which was found to have many toxic effects on the environment [9]. The high activity of Ag NPs arises from their ultra-small size and high mobility [8]. However, mobile Ag NPs are found to aggregate easily in the medium that alters their cytotoxicity, [10] and therefore, numerous studies have been conducted to improve their dispersion. To overcome these disadvantages of Ag NPs, their immobilization onto various supporting materials such as metal oxides, activated carbon, graphene oxide, polymers, etc., have been investigated [11]. Among various hybrid NPs, TiO_2_ and TNTs modified with Ag NPs are being researched for their excellent antibacterial properties both in the presence and absence of light. The modification of TiO_2_/TNTs with Ag NPs results in changes in the physicochemical characteristics such as size, shape, stability, and oxidation state of Ag NPs, which results in enhanced antibacterial, photocatalytic, and catalytic activities [12,13,14,15,16]. Wang et al. [17] observed improved and long-lasting antibacterial activity for Ag–polydopamine–TiO_2_ nanotube composites which was attributed to the tethering of Ag NPs onto TNTs by polydopamine layers.

Important factors which affect the bactericidal activity of Ag NPs are their size, shape, surface functionalization, and stability [11]. The antibacterial properties of Ag NPs are found to increase with a decrease in their diameter, and the direct interaction of Ag NPs with bacteria mainly occurs when the diameters are around 1–10 nm [18,19]. Kubacka et al. [20] synthesized Ag/TiO_2_ nanocomposites through impregnation and photodeposition methods with various Ag contents and studied their photocatalytic disinfection ability against *Escherichia coli*. They observed that below 1 wt.% of Ag, the disinfection activity of the samples obtained by the two methods was comparable while at higher silver content, the photo-deposited samples displayed improved performance. Also, they found that the presence of Ag NPs helps to improve the adhesion of bacteria onto nanocomposite surfaces and the Ag lixiviation can be controlled by optimizing the amount of Ag in the nanocomposite. Similar results were reported by other researchers [7,8]. It was also found that the Ag NPs changed the surface characteristics of TiO_2_ such as the point of zero charge (PZC), which can influence bacterial attraction to the Ag/TiO_2_ surface [20]. Es-Souni et al. [21] prepared Ag/TiO_2_ nanocomposite coatings through a sol-gel approach and found that bactericidal actions rely on Ag^+^ ion release, Ag NPs size, and hydrophilicity of the nanocomposites. Keleher et al. [22] observed higher antibacterial activity for Ag/TiO_2_ than that of Ag metal, which was ascribed to the more available surface for Ag^+^ ion release in solution. Sotiriou et al. [23] studied the leaching of Ag^+^ ions from Ag/SiO_2_ nanocomposites and reported that the amount of the released Ag^+^ corresponded to the dissolution of 1–2 silver oxide monolayers present on the surface of Ag NPs, depending on their size. The authors also found that the reduction of silver oxide to metallic silver resulted in a significant minimization of Ag^+^ ion leaching which was found to decrease the antibacterial activity against *E. coli*. The investigation on TNT ions exchanged with various metal ions presented by Rónavári et al. [24] revealed that only TNTs containing silver exhibited potential antibacterial and antifungal properties against different microbial species, which was ascribed to the release of ionic Ag^+^ to the surrounding solution. In other research [14], it was observed that the controlled release of Ag^+^ from Ag/TNTs nanocomposite through diffusion and osmosis effects provided extended antibacterial activities of this material. Rodríguez-González et al. [16], based on their research on antifungal properties of Ag/TNTs, concluded that due to the nanotubular morphology, the TNTs could easily damage cell walls and accelerate vacuolation and invagination which results in inactivation of fungi.

Silver NPs are predominantly synthesized from silver nitrate (AgNO_3_) and silver acetate (CH_3_COOAg) as the precursors [14,15,16,25]. Various reported methods for the preparation of Ag/TiO_2_ nanocomposites are photoreduction [13], sol-gel [26], chemical reduction [27], template induced and solvothermal [28] methods. The Ag/TNTs are obtained through photoreduction [29], chemical reduction [30], ion exchange followed by calcination [31], microwave-assisted methods [32], and hydrothermal processes [33]. The Ag/TNTs nanocomposites are mainly used as antibacterial nanomaterial [14], visible light photocatalyst [34], nanofiller in modified polymeric membranes [35], and as a multicolor photochromic material [36]. During the preparation of Ag-modified nanocomposites through a photoreduction approach, the physicochemical properties like size, uniformity, and density of photodeposited Ag NPs are depended upon solvent, silver precursor concentration, reaction atmosphere (ambient or inert), irradiation wavelength, and time or type of support (e.g., TiO_2_, TNTs, etc.) used [13,37,38]. Ma et al. [30] reported that Ag NPs in the metallic form with a size range of 3–10 nm could be deposited onto TNTs by NaBH_4_ reduction, whereas the reduction reaction carried out without the use of TNTs resulted in the formation of highly agglomerated Ag NPs with a size of 20–50 nm. The stability of the Ag NPs on TNTs was attributed to the strong bonding interaction between Ag NPs and the oxygen atoms of TNTs [30]. Priya et al. [27] demonstrated the synthesis of Ag_2_O/Ag^0^-loaded TiO_2_ NPs by an electroless coating technique in which Ag^+^ ions were reduced onto the TiO_2_ using Sn^2+^. Lai et al. [39] produced Ag/TiO_2_ nanotubes by hydrothermal treatment of the sol-gel-processed Ag/TiO_2_ NPs. The reduction of Ag^+^ ions to metallic Ag occurred during the thermal treatment step of the sol-gel processed TiO_2_ nanopowder. After the hydrothermal treatment, Ag NPs of sizes 4–8 nm were found to be well dispersed on the exterior of the nanotube surface with a small fraction of Ag NPs encapsulated in the interior of the TiO_2_ nanotubes [39]. To the best of our knowledge, there are no reports on the application of the electroless technique to fabricate Ag-modified TNTs.

The physicochemical and antibacterial properties of Ag-modified TNTs are expected to vary with the adopted synthesis method. In view of this, the present study is focused on the evaluation of the influence of the preparation procedure on the properties and stability of Ag-modified titanate nanotubes (Ag/TNTs). Twelve types of Ag/TNTs were prepared with different Ag incorporation approaches and Ag contents. The synthetic procedures included (i) sol-gel followed by a hydrothermal process; (ii) photodeposition under ambient conditions; (iii) photodeposition under an inert atmosphere; (iv) NaBH_4_ reduction; and (v) an electroless deposition process after activation of the TNTs’ surface with various amounts of Sn^2+^ ions. The physicochemical properties of the hybrid Ag/TNTs were examined and discussed in detail. Moreover, the antibacterial performance of the composites against both Gram-positive and Gram-negative bacteria were evaluated under dark conditions.

## 2. Materials and Methods

### 2.1. Materials

Titanium(IV) isopropoxide (TTIP, Sigma–Aldrich, St. Louis, MO, USA, 97%) and anatase TiO_2_ powder were purchased from Sigma–Aldrich Chemicals (St. Louis, MO, USA). HCl (35–38 wt.%), H_2_SO_4_ (96 wt.%), AgNO_3_, SnCl_2_, ammonia solution (25%), NaOH, Na_2_HPO_4_, and KH_2_PO_4_ were purchased from Avantor Performance Materials (Gliwice, Poland). NaBH₄ was supplied by Merck, (Darmstadt, Germany). 2-propanol, KCl, and NaCl were provided by Chempur (Piekary Śląskie, Poland).

Microbiological tests were carried out using Plate Count Agar (PCA) and Brain Heart Infusion (BHI) Agar (BIOMAXIMA, Lublin, Poland). Gram-negative *Escherichia coli* (strain K12, ATCC 29425, Manassas, VA, USA) and Gram-positive *Staphylococcus epidermidis* (ATCC 49461, Manassas, VA, USA) were used as model microorganisms. The initial concentration of bacteria suspension was set at 0.5 using McFarland scale (McFarland standards, bioMérieux, Marcy-l’Étoile, France).

In all experiments, pure (deionized) water (type 2, 0.066 µS cm^−1^) from Elix 3 (Millipore, Burlington, MA, USA) was used, unless otherwise stated.

### 2.2. Preparation of TNTs

Titanate nanotubes were prepared by employing alkaline hydrothermal treatment of anatase TiO_2_ powder. Initially, TiO_2_ (2 g) was ultrasonicated with 60 mL of 10 M NaOH solution for 1 h at room temperature to obtain a homogeneous dispersion. The mixture was then transferred to a Teflon-lined stainless-steel autoclave and then heated at 140 °C for 24 h. After being cooled down to room temperature, the product was first washed with 2 L of 0.1 M HCl and then with deionized water until the conductivity of the filtrate became ~1 µS·cm^−1^. Finally, the white product was dried at 80 °C in an oven for 12 h and stored.

### 2.3. Preparation of Hybrid Ag/TNTs

#### 2.3.1. Preparation of Ag/TNTs by Sol-Gel Combined with Hydrothermal Process

Silver-modified nanocrystalline TiO_2_ powders were synthesized by sol-gel process. First, 5.53 g of TTIP was dissolved in 100 mL of 2-propanol. A second solution was prepared by dissolving 50.96 and 254.8 mg of AgNO_3_ (corresponding to Ag: Ti atomic ratios of 0.01 and 0.05, respectively) in a mixture of deionized water (50 mL) and 2-propanol (100 mL). Both solutions were sealed immediately and stirred thoroughly using the magnetic stirrer. The water part of the solution was then added drop-wise to the alkoxide part under continuous magnetic stirring. After the complete addition of the water part of the solution, the resulting suspension was stirred for 4 h before drying in an oven at 80 °C for the complete removal of residual water and the solvent. The dried powder was then ground well using a mortar and pestle and then calcined in a muffle furnace at 500 °C for 2 h at the heating rate of 5 °C min^−1^ for the crystallization of amorphous TiO_2_. Such obtained Ag/TiO_2_ powders containing various amount of Ag were then hydrothermally treated according to the procedure described above (see Section 2.2). The resulting hybrid products were denoted as Ag/TNT-1_SH and Ag/TNT-5_SH, where the “1” and “5” represented the initially used atomic ratio of Ag/Ti for the sol-gel synthesis of Ag/TiO_2_ (Table 1).

#### 2.3.2. Preparation of Ag/TNTs by Photodeposition

In this technique, 0.5 g of TNTs were dispersed into 50 mL of AgNO_3_ solution (2.5 and 100 mM) with magnetic stirring (250 rpm) for 2 h in a glass reactor. The processes were carried out under either an ambient or inert (Ar) atmosphere. Afterward, the slurry was irradiated with a low-pressure mercury vapor lamp (TNN 15/32, Heraeus Noblelight GmbH, 15 W, λ_max_ = 254 nm) for 2 h with continuous stirring. The suspension was then collected by centrifugation and subsequently washed several times with deionized water for the complete removal of excess of Ag^+^ ions. Finally, the products were dried at 80 °C in an oven for 12 h and stored. The samples were denoted later as Ag/TNT-2.5_AM and Ag/TNT-100_AM for ambient atmosphere, or Ag/TNT-2.5_IN and Ag/TNT-100_IN for inert atmosphere, where the numbers represented the concentration of AgNO_3_ solution (Table 1).

#### 2.3.3. Preparation of Ag/TNTs by NaBH_4_ Reduction

The Ag^+^ ions were reduced onto TNTs according to the procedure described elsewhere [30]. In a typical synthesis, 0.5 g of TNTs were dispersed into 50 mL of AgNO_3_ solution (2.5 and 100 mM) and magnetically stirred for 2 h. The nanotubes were then separated from the solution by centrifugation at 3000 rpm. An ice-cold solution of NaBH_4_ (5 mL, 0.1 M) was added drop-wise to the centrifuged sample. The product was then collected and washed with deionized water before drying at 80 °C in an oven for 12 h. The samples were denoted later as Ag/TNT-2.5_NB and Ag/TNT-100_NB, where the numbers represented the concentration of AgNO_3_ solution (Table 1).

#### 2.3.4. Preparation of Ag/TNTs by Electroless Reduction

First, 0.5 g of TNTs were dispersed in 30 mL of deionized water. Then, a second solution was prepared by dissolving SnCl_2_ (either 0.1 g or 1 g) in 20 mL of 0.2 M HCl. The two solutions were then mixed and stirred for 2 h at room temperature to obtain the surface sensitized TNTs. The suspension was subsequently centrifuged at 3000 rpm and washed three times with deionized water. The residue was then transferred to 50 mL of AgNO_3_ solution (2.5 and 100 mM) and the resulting solution was made alkaline by the addition of 5 drops of aqueous NH_3_ solution. The suspension was stirred for 1 h and the product was separated by centrifugation at 3000 rpm. After washing with deionized water, the nanomaterial was dried at 80 °C in an oven for 12 h. The samples were denoted later as Ag/TNT-2.5_EL (0.1) and Ag/TNT-100_EL (0.1) (for the samples processed using 0.1 g SnCl_2_), and Ag/TNT-2.5_EL (1) and Ag/TNT-100_EL (1) (for the samples processed using 1 g SnCl_2_), where the numbers 2.5 and 100 represented the concentration of AgNO_3_ solution (Table 1).

### 2.4. Characterization Methods

The morphological analysis of pure TNTs and Ag/TNTs was carried out using a transmission electron microscope (TEM), FEI Tecnai F20. The elemental composition of the samples was studied with the usage of energy dispersive X-ray spectroscopy (EDS). The samples were prepared by sonication in ethanol followed by adding a drop of the suspension on a carbon-coated copper grid (300 mesh). The phase composition of the pure TNTs and Ag/TNTs was determined based on the X-ray diffraction (XRD) method (PANalytical Empyrean X-ray diffractometer) using CuKα radiation (λ = 1.54056 Å). Raman spectra were recorded with a 532 nm laser line (E_laser_ = 1.58eV) with a Renishaw in Via Raman micro-spectrometer. The isoelectric point (IEP) of the Ag/TNTs nanocomposites was measured using Zetasizer Nano-ZS (Malvern Instruments Ltd. Malvern, UK) equipped with a Multi-Purpose Titrator MPT-2 and a degasser. The samples were dispersed in ultrapure water and the pH was adjusted using HCl and NaOH solutions.

The composition of the Ag/TNTs surface was analyzed with use of the X-ray photoelectron spectroscopy (XPS). Measurements were conducted with Al *K_a_* (h = 1486.6 eV) radiation in a Prevac system equipped with Scienta SES 2002 electron energy analyzer operating at constant transmission energy (*E_p_* = 50 eV). The spectrometer was calibrated using the following photoemission lines (with reference to the Fermi level): EB Ag 3d_5/2_ = 368.3 eV and EB Au 4f_7/2_ = 84.0 eV. The analysis chamber was evacuated to the pressure below 1∙10^−9^ mbar. A powdered sample of the material was placed on a stainless-steel sample holder. The quantitative analysis of the surface composition was done on the basis of the peak area intensities using the sensitivity factor approach and assuming homogeneous composition of the surface layer.

Inductively coupled plasma optical emission spectrometry (ICP-OES) analysis was carried out using an Optima 5300DV spectrometer (Perkin Elmer, Waltham, MA, USA). To determine the real load of Ag in the Ag/TNTs, the samples were prepared by dissolution in a hot solution of (NH_4_)_2_SO_4_ in concentrated H_2_SO_4_. After the solution cooled down, it was diluted with water.

To determine the release kinetics of Ag^+^ ions from the different Ag/TNTs for seven days, 0.1 g of each nanocomposite was dispersed into 100 mL of deionized water and placed in a digital shaking water bath maintained at 30 °C. A defined number of samples was withdrawn after 1, 3, and 7 days and separated through a 0.2 μm filter. The concentration of Ag in the filtrate was analyzed using ICP-OES spectrometer and the given values were a mean from three repetitions.

### 2.5. Microbiological Study

#### 2.5.1. Preparation of Culture Medium

First, the BHI and PCA solutions were prepared according to the instructions given by the manufacturer. Next, the Petri dishes were filled with an adequate solution and left to be solidified. Finally, the prepared agar plates were sterilized under UVC light for 20 min and then dried in the incubator for 3 days.

A NaCl solution was prepared by dissolving 8.5 g NaCl in 1 L of distilled water and then autoclaved.

Phosphate buffered saline (PBS, pH 7.2) was obtained by dissolution of 8 g NaCl, 0.2 g KCl, 1.44 g Na_2_HPO_4_, and 0.24 g of KH_2_PO_4_ in 1 L of distilled water, and the pH was adjusted using HCl. Before application, the solution was sterilized by autoclaving.

#### 2.5.2. Antibacterial Study of Nanomaterials

A series of glass bottles filled with 100 mL of nanomaterial suspension (20 mg L^−1^) in NaCl or PBS solutions containing *E. coli* or *S. epidermidis*, respectively, were prepared. The number of bacteria was set at 0.5 according to the McFarland scale. The control sample was prepared in the same way, but without addition of NPs. The bottles were incubated for 24 h at 37 °C with continuous stirring at 250 rpm. After that, the bacteria were counted using the serial decimal dilutions in NaCl and PBS solutions, respectively. 0.3 mL of a suitable diluted solution was put in the middle of a plate containing PCA or BHI and spread using a spreader. Three repetitions for each dilution were prepared. The plates with bacteria were incubated at 37 °C for 24 h. After that, the visible colonies of bacteria on agar plates were calculated by the counter (LKB 2002, POL-EKO, Wodzisław Śląski, Poland). The average colony forming unit (CFU) per mL values were evaluated according to Equation (1):

(1) $$\mathrm{CFU/mL}=\frac{N\times Y}{Z}$$

where: *N*—number of bacteria colonies visible on the Petri dish, *Y*—total dilution factor, and *Z*—volume of bacteria suspension put on the agar plate (0.3 mL).

The log reduction of bacterial growth was determined with reference to the blank sample using Equation (2):

(2) $$\mathrm{log reduction}=\log\left(\frac{A}{B}\right)$$

where: *A*—number of bacteria determined in control sample, i.e., without addition of NPs (CFU/mL), *B*—number of bacteria determined in the presence of NPs (CFU/mL).

## 3. Results and Discussion

### 3.1. ICP Compositional Analysis

The amount of Ag in the different Ag/TNTs nanocomposites was evaluated based on ICP-OES analysis, and the values are presented in Table 2.

For all preparation procedures, the weight fraction of Ag in the nanocomposite was found to be increased with an increase in the initial concentration of AgNO_3_. A minimum Ag content was observed for Ag/TNT-1_SH, and a maximum Ag loading was found in the case of Ag/TNT-100_EL (1). The amount of Ag was almost comparable for photodeposition (both inert and ambient atmosphere), and NaBH_4_ reduction processes, with a moderately higher Ag loading observed for the latter approach. Silver/TNTs processed using an electroless method indicate the presence of Sn, originating from the SnCl_2_ used as a reducing agent. The amount of Sn in the case of Ag/TNT-2.5_EL was found to be higher compared to Ag/TNT-100_EL samples for both 0.1 g and 1 g SnCl_2_ loading, which confirms the role of Sn^2+^ ions in the reduction of Ag^+^ to Ag^0^ on the TNTs.

### 3.2. Morphological Analysis

**Figure 1:**
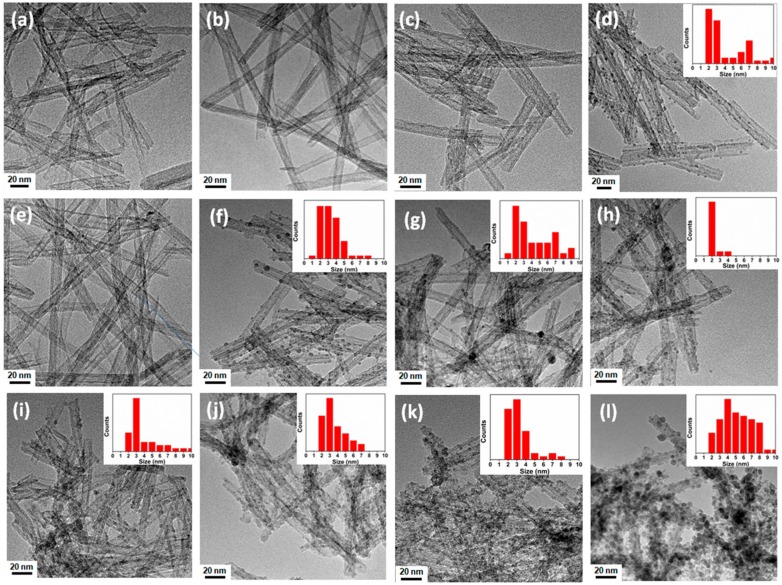
TEM images of Ag/TNTs and size distribution (inserted) of Ag nanoparticles (NPs) on TNTs: (**a**) Ag/TNT-1_SH, (**b**) Ag/TNT-5_SH, (**c**) Ag/TNT-2.5_AM, (**d**) Ag/TNT-100_AM, (**e**) Ag/TNT-2.5_IN, (**f**) Ag/TNT-100_IN, (**g**) Ag/TNT-2.5_NB, (**h**) Ag/TNT-100_NB, (**i**) Ag/TNT-2.5_EL (0.1), (**j**) Ag/TNT-100_EL (0.1), (**k**) Ag/TNT-2.5_EL (1), (**l**) Ag/TNT-100_EL (1).

Transmission electron microscopy was employed in order to determine the morphology of the Ag/TNTs and the size distribution of Ag NPs deposited on TNTs. The results are presented in Figure 1.

The TEM images confirmed the formation of open-ended nanotubes with lengths in the range of ~50–200 nm and diameters of ~5–10 nm for all the Ag/TNTs. The sol-gel-assisted hydrothermal method of TNT modification did not result in the formation of Ag NPs within the used concentrations of the AgNO_3_ solution. In the case of the photodeposition approach, the presence of the Ag NPs was observed when higher concentrations of AgNO_3_ solution were applied, regardless of the atmosphere of the reaction. The ICP-OES compositional measurement (Table 2) also indicated low content of Ag for Ag/TNT-1_SH, Ag/TNT-5_SH, Ag/TNT-2.5_AM, and Ag/TNT-2.5_IN. Electroless and NaBH_4_ reduction methods led to the creation of Ag NPs in the case of both lower and higher AgNO_3_ solution concentration. The particle size distribution data in the insets in Figure 1d,f–l indicate the presence of NPs with sizes between 1–10 nm on TNTs. The sizes ranging from 2 to 10 nm, 1 to 8 nm, 1 to 9 nm and 2 to 4 nm corresponded to Ag NPs present in Ag/TNT-100_AM, Ag/TNT-100_IN, Ag/TNT-2.5_NB, Ag/TNT-100_NB, respectively. In the case of Ag/TNT-2.5_EL (0.1), Ag/TNT-100_EL (0.1), Ag/TNT-2.5_EL (1), and Ag/TNT-100_EL (1), the determined particle size (2 to 10 nm, 2 to 7 nm, 2 to 8 nm, and 2 to 10 nm, respectively) refers to both Ag and SnO_2_ identified on the surface of the samples processed by the electroless method.

The TEM images also demonstrate the difference between physical and chemical Ag deposition processes (Supplementary Materials, Figure S1). The photoreduction approach led to creation of Ag NPs exclusively on the outer surface of TNTs, whereas chemical reduction techniques introduced Ag NPs both on the outer surface and inside the TNTs. This is because during UV irradiation, the outer region of the TNTs was more exposed to the action of the radiation, and hence, Ag^+^ ion reduction preferably occurred in this area as observed in Figure S1a. For chemical reduction, the reducing agent had equal accessibility for the exterior and interior surfaces of the TNTs [40]. Hence, the Ag NPs could be deposited on either side of the TNTs as observed in Figure S1b,c. From particle size distribution analysis, it was observed that Ag/TNT-100_NB (inset of Figure 1h) contained almost exclusively Ag NPs with a size of 2 nm. The small size of the Ag NPs helped in the modification of both the inner and outer sides of the TNTs for this sample. However, for electroless deposition process, both surfaces of TNTs were found to be covered with an excess of NPs. This was due to the coexistence of both Ag and SnO_2_ NPs with similar size making them indistinguishable from each other.

Based on TEM-EDS analysis (Figure S2), it was found that Ag/TNT-2.5_AM (Figure S2a) was characterized by a homogenous distribution of Ag all over the TNTs, despite the absence of NPs, as was found from Figure 1c. This suggests that Ag was built into the structure of nanotubes. In the case of Ag/TNT-5_SH (Figure S2b), an additional signal corresponding to Cl can be noticed, which indicates the presence of AgCl, possibly formed during the acid (HCl) washing step after the hydrothermal treatment. The EDS elemental mapping of Ag/TNT-2.5_EL (1), shown in Figure S2c, reveals the presence of Sn (red color) in addition to Ag (blue color). It can also be observed that Sn was uniformly distributed all over the TNTs in contrast to much smaller amounts of Ag concentrated in particular places. It is clear from the high resolution TEM (HRTEM) image of Ag/TNT-100_EL (1) shown in Figure S3 that the Ag and SnO_2_ NPs were attached to the TNTs, and the interplanar spacing of NPs with distances 0.23 nm and 0.34 nm could be attributed to the (111) planes of Ag and (110) planes of SnO_2_, respectively. Hence, the application of the electroless process resulted in the modification of TNTs with both Ag and SnO_2_ NPs. From Table 2, it was observed that the Ag/TNT-2.5_EL (0.1) and Ag/TNT-2.5_EL (1) exhibited higher concentrations of Sn compared to Ag, therefore, it can be concluded that majority of NPs on the surface of TNTs for these materials are SnO_2_ NPs.

### 3.3. XRD Analysis

**Figure 2:**
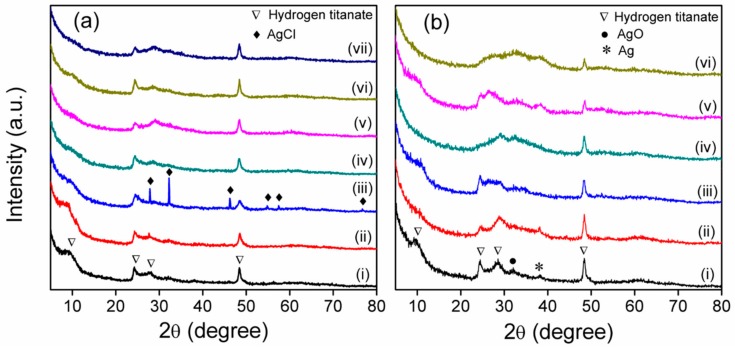
(**a**) XRD pattern of (i) pure TNTs, (ii) Ag/TNT-1_SH, (iii) Ag/TNT-5_SH, (iv) Ag/TNT-2.5_AM, (v) Ag/TNT-100_AM, (vi) Ag/TNT-2.5_IN, (vii) and Ag/TNT-100_IN. (**b**) XRD pattern of (i) Ag/TNT-2.5_NB, (ii) Ag/TNT-100_NB, (iii) Ag/TNT-2.5_EL (0.1), (iv) Ag/TNT-100_EL (0.1), (v) Ag/TNT-2.5_EL (1), and (vi) Ag/TNT-100_EL (1).

The structural evolution from pure TNTs to different Ag/TNTs was studied by XRD measurements (Figure 2).

Figure 2a (i) shows the XRD pattern of pure TNTs, and it exhibited peaks at *2θ*~9.7°, 24.3°, 27.8°, and 48°, which indicate the formation of layered titanates such as H_2_Ti_2_O_5_⋅H_2_O, H_2_Ti_3_O_7_ or H_x_Ti_2−x/4_□_x/4_O_4_ (x~0.7, □: vacancy) [31,41,42]. In comparison with pure TNTs, noticeable structural changes were observed in the XRD pattern of Ag/TNTs especially at higher Ag loading. The most perceptible change with Ag loading is the disappearance of the peak at *2θ*~9.7° that resulted from the (100) plane of the TNTs. This was because during the synthesis of Ag/TNTs, Ag^+^ ions first diffused to the TNTs’ surface and deposited as silver hydrate intermediate (Ag(OH)_n_(H_2_O)_m_), which upon dehydration with surface Ti–OH groups resulted in binding to the surface by sharing with surface oxygen atoms of the TiO_6_ octahedron layers in the (100) planes of the TNTs. As a result, the (100) planes of the TNTs underwent drastic deformation, and hence loss of its X-ray diffraction pattern with Ag loading [43]. In addition, the X-ray diffraction peak at *2θ*~24.3° was found to be distorted or weakened with Ag incorporation, which also resulted from the deformation at the surface of crystal lattice caused by the modification of the layered titanate structure [44]. This effect is maximal for Ag/TNT-100_NB, Ag/TNT-100_EL (0.1), Ag/TNT-2.5_EL (1), and Ag/TNT-100_EL (1). This is due to the presence of a high concentration of Ag and/or SnO_2_ NPs, as shown by ICP-OES (Table 2) and TEM (Figure 1) analysis.

The XRD patterns of Ag/TNTs processed from sol-gel-derived Ag modified anatase TiO_2_ (Ag/TNT-1_SH and Ag/TNT-5_SH) indicate the presence of AgCl (JCDPS 31-1238). The EDS mapping also suggested the presence of Cl in Ag/TNT-5_SH (Figure S2b). Furthermore, a higher fraction of Ag was found to be transformed to AgCl for Ag/TNT-5_SH compared to Ag/TNT-1_SH, which was directly related to the Ag content of the Ag–TiO_2_ precursor used for hydrothermal treatment. The analysis of Ag/TNTs processed through electroless approach and NaBH_4_ reduction method revealed two spikes at 2*θ*~32° and 2*θ*~38°, which can be assigned to silver oxide (AgO) (JCDPS 76-1489) and elemental Ag (JCDPS 04-0783), respectively.

### 3.4. Raman Spectra Analysis

**Figure 3:**
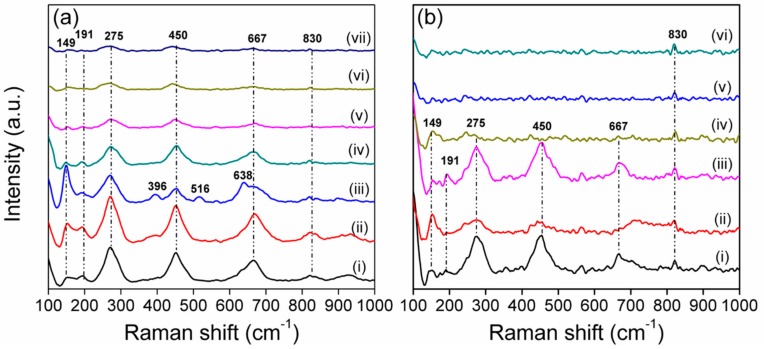
(**a**) Raman spectra of (i) pure TNTs, (ii) Ag/TNT-1_SH, (iii) Ag/TNT-5_SH, (iv) Ag/TNT-2.5_AM, (v) Ag/TNT-100_AM, (vi) Ag/TNT-2.5_IN, and (vii) Ag/TNT-100_IN. (**b**) Raman spectra of (i) Ag/TNT-2.5_NB, (ii) Ag/TNT-100_NB, (iii) Ag/TNT-2.5_EL (0.1), (iv) Ag/TNT-100_EL (0.1), (v) Ag/TNT-2.5_EL (1), and (vi) Ag/TNT-100_EL (1).

Figure 3 shows the Raman spectra of the prepared TNTs and Ag/TNTs. Almost identical Raman vibration patterns were observed for both TNTs and Ag/TNTs, which consisted of mainly four very broad bands centered at 275, 450, 667, and 830 cm^−1^ that could be assigned to the protonated type of TNTs [34,45,46,47]. The occurrence of the peak at 149 cm^−1^ indicates the formation of a tetrahedron structure in the nanotubes with oxygen deficiencies [48]. The presence of Raman bands at 191, 275, 450, 667, 830, and 930 cm^−1^ confirmed the formation of H_2_Ti_3_O_7_ nanotubes [41,49,50]. According to previous reports, the three Raman bands at around 270, 450, and 700 cm^−1^ are assigned to the A_g_ symmetric modes of Ti–O–Ti vibrations of layered titanates [41]. The bands at 830 cm^−1^ are assigned to the Ti–O–H symmetric stretching mode with short Ti–O distance [51], and the band at 930 cm^−1^ is due to the four coordinate Ti–O vibrations in the titanate structure [52]. Raman spectra of Ag/TNT-100_NB, Ag/TNT-100_EL (0.1), Ag/TNT-2.5_EL (1). and Ag/TNT-100_EL (1) also showed a loss characteristic vibration of TNTs due to the higher loading of NPs (both Ag and SnO_2_). This indicate that higher metal loading drastically alters and/or diminishes the characteristic XRD and Raman vibration pattern of TNTs.

Different from the other samples, the spectra of Ag/TNTs prepared by sol-gel-assisted hydrothermal process show low intensity peaks at 396, 516, and 638 cm^−1^ which correspond to B_1g_, A_1g_, and E_2g_ vibration modes of anatase TiO_2_, respectively [53]. Additionally, the intensity of the band at 149 cm^−1^ was found to be comparatively higher for this Ag/TNTs. This indicates that a small fraction of anatase TiO_2_ remained unconverted after the hydrothermal process for these samples [32,49]. However, the presence of anatase TiO_2_ could not be confirmed based on the XRD analysis (Figure 2) due to (i) the overlapping of the anatase TiO_2_ peak with that of hydrogen titanate or (ii) too low content of anatase to be detected by this method.

Based on the XRD and Raman analyses, it can be concluded that the hydrogen titanate structure of Ag/TNTs remains unaffected at lower Ag loading and is affected when the Ag loading is higher.

### 3.5. XPS Analysis

The surface concentration of elements was measured with application of XPS analysis. Silver/TNT samples prepared by various methods using higher AgNO_3_ content were selected (Table 3). The surface of all these samples consisted of titanium, oxygen, silver, and carbon atoms. The presence of Sn atoms was proven for the samples prepared by electroless reduction, Ag/TNT-100_EL (0.1) and Ag/TNT-100_EL (1). Sodium atoms were present on the surface of Ag/TNT-100_NB sample, which was confirmed by the Na KLL Auger peak. Unfortunately, the XPS Na 1s and Auger Ti LMM lines overlapped. Therefore, the XPS Na 1s peak’s intensity could not be resolved and the concentration of sodium atoms was not considered in the calculation of the surface composition of Ag/TNT-100_NB sample.

In Table 3, the surface composition of the samples analyzed by XPS is shown. The calculations were employed with the assumption that the spatial distribution of all elements identified in a near-surface region was homogeneous.

In general, the Ti:O ratio observed for the investigated samples was close to 1:3. Therefore, the surface structure of Ti–O compounds was considered as a TiO(OH)_2_ type rather than TiO_2_ type. In samples Ag/TNT-100_EL (0.1) and Ag/TNT-100_EL (1), the Ti:O ratio was even smaller due to a significant concentration of oxygen atoms being a part of Sn–O compounds. Since the depth of detection for XPS was around 5–10 nm [54,55], the Ag NPs present on both the outer and inner TNTs walls were analyzed (Figure S1). This led to the similar Ag concentrations for Ag/TNT-100_AM, Ag/TNT-100_IN, and Ag/TNT-100_NB (2–3 at.%). A significant enrichment of the surface with silver atoms was observed for the samples obtained by the electroless reduction, especially in the sample Ag/TNT-100_EL (1). Considering that the surface concentration of silver w presented in atomic percent, the direct correlation of these data with the silver concentrations measured by ICP-OES method is not possible. However, a general relation of these concentrations between the samples is kept (Figure S4).

The high-resolution XPS spectra were analyzed to elucidate the chemical state of silver atoms formed by different types of preparation methods. The XPS Ag 3d spectra (Figure S5) have virtually identical positions of the maximum and a very symmetric spectrum envelope. They contained two spin-orbit components: 3d_5/2_ and 3d_3/2_ located at the binding energy of 368.3 eV and 374.3 eV, respectively. The full-width at half maximum (FWHM) of these components was also identical for all samples and amounted to 1.7 eV. Therefore, it is concluded that the chemical state of silver in all analyzed samples was identical. The binding energy of the maximum of XPS 3d_5/2_ component at 368.3 eV was characteristic for metallic silver [56]. The presence of silver oxides which can be considered in the context of Ag/TNTs materials should result in the XPS features located at the binding energy region between 367.3 eV and 368 eV.

### 3.6. Ag^+^ Ion Release Measurement

**Figure 4:**
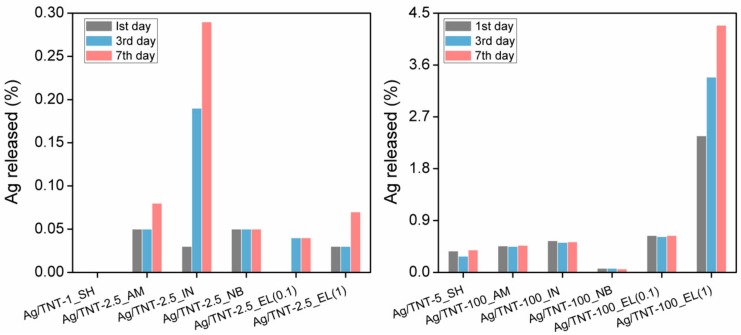
The percentage of Ag^+^ released from different Ag/TNTs.

One of the critical parameters which determines the antibacterial properties of nanocomposites modified with Ag NPs is Ag^+^ ions’ release ability [51]. Figure 4 shows the percentage of Ag^+^ released from different Ag/TNTs for a period of one, three, and seven days of immersion in deionized water. The values were calculated with reference to the initial silver content in Ag/TNTs (Table 2).

During a period of seven days, a very small amount of Ag^+^ (<5%) was observed to be leached from the prepared Ag/TNTs which indicated their high stability. The amount of Ag^+^ leaching from the NPs was found to increase with the increase in immersion time. However, for some Ag/TNTs, the amount of Ag released after the third day was slightly lower than after the first day (e.g., Ag/TNT-5_SH). This could be explained by reincorporation of the released Ag^+^ ions in the TNTs’ structure by the ion-exchange process. The minimum Ag^+^ release percentage was observed for Ag/TNT-1_SH and the maximum release exhibited the Ag/TNT-100_EL (1) which was directly related to the Ag content in the nanocomposite (Table 2). For Ag/TNTs with lower Ag content (less than 5 wt.%, Table 2), Ag/TNT-5_SH showed the highest Ag^+^ release percentage. This sample was prepared by sol-gel followed by a hydrothermal process and was characterized by the presence of AgCl (almost insoluble in water, K_sp_ for AgCl at room temperature was 1.77 × 10^−10^ [57]) except from metallic Ag. In the case of this sample, no Ag NPs were identified on the surface (Figure 1b), although a uniform distribution of silver was confirmed by the TEM-EDS mapping (Figure S2b). Therefore, its low stability can be attributed to a dissolution of a silver layer covering the TNTs, a release of silver ions from AgCl or a removal of Ag^+^ from titanate structure, where it was possibly built-in via the ion exchange process during the synthesis step. Except for Ag/TNT-100_NB, in the cases of all the other Ag/TNTs processed using a higher concentration of AgNO_3_ (100 mM), the Ag^+^ release percentage was directly related to the initial Ag content. The sample Ag/TNT-100_NB exhibited the maximum stability for Ag^+^ leakage during the seven days of measurement which could be attributed to the presence of Ag NPs not only on the outer surface of the TNTs but also inside the nanotubes, as observed in Figure S1c–f. Such a structure can be regarded as a container (TNT) housing Ag NPs and serving as protection which hindered Ag^+^’s release.

### 3.7. Surface Charge Measurements

On the basis of the zeta potential measurement as a function of pH, the isoelectric point (IEP) of the NPs was evaluated, and the corresponding values are summarized in Table 4.

Zeta potential describes the electrostatic interactions between the charged surface of a particle and the bulk of a liquid. There is an electrical double layer surrounding the particle. In the inner layer, the ions are strongly bound to the particle, and in the outer layer (diffuse layer) they are less firmly attached. The boundary between those regions is called the shear or slipping plane. The zeta potential is the potential between the dispersion medium and the stationary layer of the fluid attached to the particle. The zeta potential strongly depends on pH [58]. The pH at which the surface of the NPs has zero net charge is called the IEP [59]. When the pH is above the IEP, the surface sites become negatively charged either by adsorbing hydroxyl ions or by desorbing protons and vice versa. The value of the zeta potential of the nanomaterials could also affect their interactions with other species, such as microorganisms present in a liquid. The IEP of living *E. coli* and *S. epidermidis* are around 2.4 and 1.5–2.0, respectively [60,61]. Thus, the bacteria cells have positive charge only under very acidic conditions.

Both pure TNTs and Ag/TNTs show IEP in the range of ~3.1–3.9 and exhibit negative zeta potential above these values. The IEP of Ag/TNTs was found to be at higher pH than that of pure TNTs which could be attributed to (i) the presence of silver species, either in the form of Ag NPs or as Ag^+^ ions replacing H^+^ in TNTs structure, and (ii) the presence of SnO_2_ NPs in case of the samples prepared by the electroless method. A similar trend in zeta potential was observed when the TiO_2_ surface was modified with metals like Cu, Fe or Co [62]. However, for the same method of preparation, the Ag/TNTs with higher Ag content have slightly lower IEP values than that of Ag/TNTs with lower Ag content. This is possibly because at a high Ag amount, the role of Ag NPs, which have an IEP of ~2.5 in deionized water, becomes more prominent [63].

### 3.8. Antibacterial Properties of Ag/TNTs

**Figure 5:**
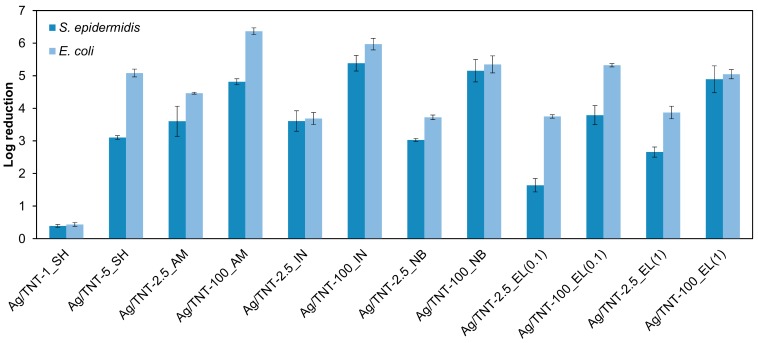
Antibacterial properties of Ag/TNTs towards *E. coli* and *S. epidermidis*.

The antimicrobial properties of various Ag/TNTs were evaluated with reference to two types of bacteria: Gram-positive (*S. epidermidis*) and Gram-negative *(E. coli*). The results are presented in Figure 5. In general, for every type of Ag/TNTs synthesis approach, the samples obtained using lower AgNO_3_ amount (i.e. 2.5 mM AgNO_3_), and thus containing lower silver loading, were less active compared to the NPs synthesized with application of higher AgNO_3_ concentration (i.e. 100 mM AgNO_3_). 

Analyzing the results shown in Figure 5, it can also be observed that *S. epidermidis* was inactivated with lower efficiency than *E. coli*, regardless of the Ag/TNTs used. This phenomenon can be related with the composition of the bacteria cell wall. Gram-positive bacteria (i.e., *S. epidermidis*) have a relatively thick (20–80 nm), continuous cell wall, composed of peptidoglycan and covalently attached to other cell wall polymers (teichoic acids, polysaccharides, peptidoglycolipids) [64]. Such a structure results in a high rigidity of the bacterial cell as well as provides a very limited number of anchoring sites for Ag NPs and makes Ag NPs and ions difficult to penetrate [65,66]. On the other hand, Gram-negative bacteria possess a thin (5–10 nm) peptidoglycan layer, which in the case of *E. coli* is probably only a monolayer thick [64]. Outside the peptidoglycan layer, there is an outer membrane (7.5–10 nm). Despite the presence of many covalent bonds between polysaccharides and lipids in the outer membrane, the strength and rigidity of Gram-negative bacteria are low. Additionally, the presence of micro-channels known as porins responsible for bilateral transport of substances can facilitate transport of Ag^+^ ions to the inner of bacteria cells [67].

The exact mechanism of action of nano-Ag as an antibacterial agent is not fully understood; however, in general, its antibacterial behavior is explained with the help of three approaches. First, due to the high binding affinity of Ag towards sulfur, Ag NPs attach to the bacterial cell membrane due to the presence of sulfur-containing proteins in it and cause many structural and functional changes to it [68,69]. Secondly, nano-Ag undergoes oxidation and the formed Ag^+^ ions are released to the physiological environment which upon complexation with nucleic acids leads to DNA condensation and loss of replication ability [69]. Also, the Ag^+^ ion has high affinity towards the thiol group of the cysteine residues of protein NADH dehydrogenases and causes disorder to the respiratory chain which finally leads to cell damage [70]. Thirdly, reactive oxygen species (ROS) like hydrogen peroxide (H_2_O_2_), hydroxyl radicals (OH^•^) or superoxide anions (^•^O_2_^−^) formed in the presence of Ag NPs also contribute to the bactericidal actions [71,72].

Based on the results presented in Figure 5, it can be observed that the method of preparation of Ag/TNTs had some influence on the antimicrobial action of the nanomaterials. Ivask et al. [73] investigated the relation between size and antibacterial activity of Ag NPs and suggested that the mechanism of action is mainly dependent upon Ag NPs size. When the Ag NP’s diameter is above 10 nm, the antibacterial activity depends on the released Ag^+^ ions, whereas when the size of Ag NP’s diameter is below 10 nm, the interaction of NPs with the bacterial cell wall becomes more important. In the present investigations, the prepared Ag/TNTs contain Ag NPs with a size <10 nm, and hence, it can be expected that the antibacterial action occurs through direct interaction of Ag NPs present in the hybrid structures with the bacterial cell walls. From the morphological analysis (Figure S1), it was noticed that the Ag/TNTs processed through the photodeposition method contained Ag NPs which were mostly attached to the outer surface of TNTs. This led to the higher antibacterial activity of nanomaterials synthesized by the photoreduction approach than that of Ag/TNTs prepared by chemical reduction methods, using both NaBH_4_ and SnCl_2_ as reducing agents, even though they contained higher amounts of Ag (Table 2). Nonetheless, in the presence of SnO_2_, the mechanism of antibacterial action of the NPs can differ from that of TNTs containing Ag only. The bactericidal potency of SnO_2_ was investigated by Vidhu and Philip [74] who found formation of zones of inhibition in the presence of these nanoparticles. They attributed that to the mechanism typical for metal oxides, i.e., formation of reactive oxygen species and electrostatic interaction of nanostructures with bacterial cell walls. Furthermore, Kumar Nair et al. [75] reported a synergic antimicrobial action of Ag and SnO_2_ towards *E. coli*. However, the authors applied UV irradiation to induce the antibacterial action. Nonetheless, the above data show that a direct comparison of Ag/TNTs containing solely Ag and those modified with both Ag and SnO_2_ NPs is difficult and the explanation of the antimicrobial action of the NPs containing tin oxide needs further investigations. Nonetheless, in general, the obtained results revealed that the efficiency of inhibition of *E. coli* and *S. epidermidis* growth was lower when SnO_2_ was present in the samples. Another mechanism of antibacterial action can be expected for the sol-gel-derived Ag/TNTs. In the case of these nanomaterials, no Ag NPs were detected during TEM analysis (Figure 1a,b); however, the XRD measurement confirmed the presence of the AgCl phase (Figure 2). Okkyoung et al. [76] in their work demonstrated that colloidal AgCl can be as important antibacterial agent as the other Ag forms, including Ag^+^ ions. Taking the above into consideration the antibacterial properties of the discussed nanomaterials can be linked with the presence of AgCl.

**Figure 6:**
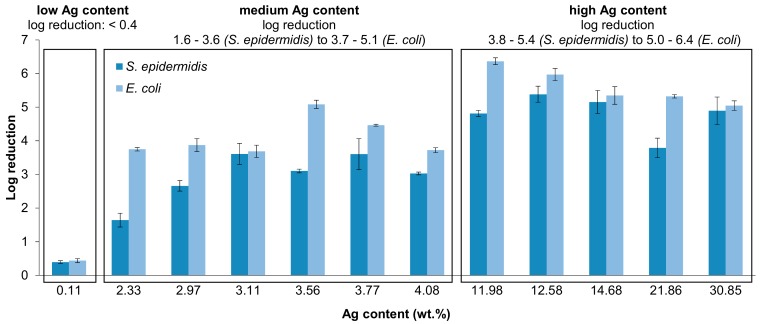
Antibacterial properties of Ag/TNTs with reference to Ag content in the hybrid nanomaterials.

In order to evaluate if there is any correlation between silver content in the hybrid Ag/TNTs and the log reduction values, the samples were divided into three groups, i.e., containing low (0.11 wt.%), medium (2.33–4.08 wt.%), and high (≥11.98 wt.%) amounts of Ag. Figure 6 summarizes the results. It can be observed that the nanomaterial with the lowest Ag content (0.11 wt.%) was characterized by the lowest antimicrobial activity against both bacteria. Analysis of the samples modified with a medium amount of Ag revealed that the antibacterial performance of the NPs containing between 2 and 4 wt.%Ag did not differ much, especially in terms of inhibition of *E. coli* growth. The highest log reduction with reference to both microorganisms (*E. coli*: 4.5 log reduction; *S. epidermidis*: 3.6 log reduction) exhibited Ag/TNT-2.5_AM, containing 3.77 wt.%Ag. A slightly better activity towards *E. coli* (5.1 log reduction) was observed in the case of Ag/TNT-5_SH (3.56 wt.%Ag); however, that sample was less efficient in terms of *S. epidermidis* inactivation (3.1 log reduction). In the discussed group of the NPs with medium Ag content, the lowest amount of silver was measured for Ag/TNT-2.5_EL (0.1) (2.33 wt.%), for which the sample exhibited the lowest activity with reference to the Gram-positive bacteria. The third group of Ag/TNTs covered samples with the highest Ag content. Amongst them, the best antibacterial performance against both *E. coli* and *S. epidermidis* revealed Ag/TNT-100_IN (6.0 and 5.4 log reduction, respectively), containing 12.58 wt.%Ag. No correlation between Ag content and antibacterial activity of the samples assigned to the third group was found. For example, the nanomaterial containing the highest Ag amount (30.85 wt.%) was less effective than the NPs with 12.58 wt.%Ag content. Such a phenomenon can be ascribed to the presence of Sn in the samples prepared by the electroless method (Table 2). Hassan et al. [77] observed that depending on SnO_2_ content, the antibacterial activity of SnO_2_/TiO_2_ composites can be improved or decreased. Therefore, as was mentioned earlier, the antibacterial properties of TNTs modified with both Ag and Sn NPs need further detailed studies.

Another attempt in explanation of antibacterial properties of the various Ag/TNTs was based on Ag^+^ release from the NPs (Figure 4). The samples were again divided into three groups, i.e., characterized by low (≤0.05% versus total Ag content or ≤0.2 mg L^−1^ in solution), medium (0.07–0.55% or 0.11–0.55 mg L^−1^) and high (≥0.64% or ≥1.13 mg L^−1^) Ag^+^ leakage (Figure S6). The lowest inhibition of bacterial growth was found for Ag/TNT-1_SH, in which case no Ag^+^ leaching for seven days was observed. However, no correlation between Ag^+^ release and antibacterial properties was noted. For example, the least stable Ag/TNT-100_EL (1), for which the concentration of the released Ag^+^ was the highest (7.32 mg L^−1^), exhibited a similar activity as Ag/TNT-100_NB with approximately 66 times lower Ag^+^ leakage (0.11 mg L^−1^).

The above analysis confirms that when the size of Ag NPs is less than 10 nm, the interaction of NPs with the bacterial cell is more important than Ag content or Ag^+^ ion release. Nonetheless, in general, the samples containing higher Ag loading were more active than samples with low silver amount. Furthermore, the nanomaterials for which silver concentration in the solution was in the medium and high range were characterized by higher antibacterial activity than those exhibiting the lowest Ag^+^ release. A comparison of the Ag/TNTs synthesis procedures analyzed in this study revealed that the photodeposition approach was the most effective technique to obtain nanomaterials with the best antibacterial properties.

## 4. Conclusions

Silver/TNTs with different Ag contents were synthesized through five different procedures including (i) sol-gel followed by a hydrothermal process; (ii) photodeposition under ambient conditions; (iii) photodeposition under an inert atmosphere; (iv) NaBH_4_ reduction; and (v) an electroless deposition process after activation of TNTs’ surface with various amounts of Sn^2+^ ions. The physicochemical characterization of various Ag/TNTs revealed the presence of Ag NPs in most samples. The NPs with size ~1–10 nm were uniformly deposited onto TNTs’ surface. Moreover, the electroless deposition resulted in the additional decoration of TNTs with SnO_2_ NPs. The presence of Ag NPs was not confirmed in case of nanomaterials obtained by the method (i), for which the AgCl phase was detected by the XRD analysis. Furthermore, no Ag NPs were observed in samples prepared from 2.5 mM AgNO_3_ solution using the photodeposition approach. Nonetheless, the ICP-OES analysis confirmed that all samples contained Ag and its loading varied from 0.11 wt.% to 30.85 wt.%. For Ag/TNTs with higher Ag content, a maximum stability of the nanocomposite was shown by the sample prepared with NaBH_4_ reduction method. It was noted that for the photoreduction process, the Ag NPs were specifically deposited on the outer surface of the TNTs while chemical reduction led to the introduction of Ag NPs on both inner and outer surfaces. The antibacterial activity of different Ag/TNTs against both Gram-positive (*S. epidermidis*) and Gram-negative (*E. coli*) bacteria was evaluated under dark conditions. In general, Ag/TNTs with higher Ag content exhibited higher antibacterial activity compared to the nanomaterials with lower Ag loading. Also, *S. epidermidis* was inactivated with lower efficiency compared to *E. coli*, regardless of the hybrid NPs used. The Ag/TNTs obtained by the photodeposition approach were found to exhibit moderately higher antibacterial properties compared to samples prepared by other methods due to the higher interaction of Ag NPs present on the TNTs with bacterial cell walls.

## Figures and Tables

**Table 1:** Summarizes the applied methods of Ag/titanate nanotube (TNT) synthesis, the corresponding concentrations of AgNO_3_, and samples nomenclature.

No.	Method of Ag/TNTs Synthesis	Amount or Concentration of AgNO_3_	Sample Name
1	Sol-gel and hydrothermal	0.01 (Ag/Ti)	Ag/TNT-1_SH
		0.05 (Ag/Ti)	Ag/TNT-5_SH
2	Photodeposition (Ambient atmosphere)	2.5 mM	Ag/TNT-2.5_AM
		100 mM	Ag/TNT-100_AM
3	Photodeposition (Inert atmosphere)	2.5 mM	Ag/TNT-2.5_IN
		100 mM	Ag/TNT-100_IN
4	NaBH_4_ reduction	2.5 mM	Ag/TNT-2.5_NB
		100 mM	Ag/TNT-100_NB
5	Electroless reduction (0.1 g SnCl_2_)	2.5 mM	Ag/TNT-2.5_EL (0.1)
		100 mM	Ag/TNT-100_EL (0.1)
6	Electroless reduction (1 g SnCl_2_)	2.5 mM	Ag/TNT-2.5_EL (1)
		100 mM	Ag/TNT-100_EL (1)

**Table 2:** Amount of Ag in different Ag/TNTs nanocomposites measured by ICP-OES.

Sample Name	Ag (wt.%)	Sn (wt.%)	Sample Name	Ag (wt.%)	Sn (wt.%)
Ag/TNT-1_SH	0.11	-	Ag/TNT-2.5_NB	4.08	-
Ag/TNT-5_SH	3.56	-	Ag/TNT-100_NB	14.68	-
Ag/TNT-2.5_AM	3.77	-	Ag/TNT-2.5_EL (0.1)	2.33	5.17
Ag/TNT-100_AM	11.98	-	Ag/TNT-100_EL (0.1)	21.86	4.67
Ag/TNT-2.5_IN	3.11	-	Ag/TNT-2.5_EL (1)	2.97	20.01
Ag/TNT-100_IN	12.58	-	Ag/TNT-100_EL (1)	30.85	14.38

**Table 3:** The surface concentration of elements identified by XPS on the surface of selected samples.

Element	Ti	O	C	Ag	Sn
Sample	at.%				
Ag/TNT-5_SH	23	73	4	traces	-
Ag/TNT-100_AM	19	63	15	3	-
Ag/TNT-100_IN	19	63	16	2	-
Ag/TNT-100_NB	18	61	18	3	-
Ag/TNT-100_EL (0.1)	16	59	15	5	5
Ag/TNT-100_EL (1)	11	60	9	9	11

**Table 4:** The isoelectric point (IEP) of the TNTs and Ag/TNTs.

Sample name	IEP	Sample name	IEP
TNTs	3.13(0.09)	Ag/TNT-100_IN	3.33(0.01)
TNTs (from sol-gel TiO2)	3.73(0.06)	Ag/TNT-2.5_NB	3.45(0.01)
Ag/TNT-1_SH	3.89(0.04)	Ag/TNT-100_NB	3.37(0.08)
Ag/TNT-5_SH	3.68(0.01)	Ag/TNT-2.5_EL (0.1)	3.39(0.01)
Ag/TNT-2.5_AM	3.39(0.08)	Ag/TNT-100_EL (0.1)	3.14(0.02)
Ag/TNT-100_AM	3.29(0.04)	Ag/TNT-2.5_EL (1)	3.69(0.03)
Ag/TNT-2.5_IN	3.38(0.03)	Ag/TNT-100_EL (1)	3.27(0.02)

## Supplementary Materials

The following are available online at https://www.mdpi.com/2079-4991/9/5/795/s1, Figure S1: HRTEM image of (a) Ag/TNT-100_IN, (b) Ag/TNT-100_EL (0.1), and (c)–(f) Ag/TNT-100_NB (The circled regions in (e) and (f) represent the Ag NPs anchored to the inner surface of the TNTs. Moreover, in Figure S1(e) the Ag NP blocking the entrance to the TNT can be observed), Figure S2: EDS elemental mapping of (a) Ag/TNT-2.5_AM, (b) Ag/TNT-5_SH, (c) Ag/TNT-2.5_EL (1). Scanning transmission electron microscopy (STEM) images with red squares present the scanned area, Figure S3: HRTEM image of Ag/TNT-100_EL (1), Figure S4: The dependence between Ag content measured by XPS and ICP methods, Figure S5: XPS spectra of (i) Ag/TNT-5_SH, (ii) Ag/TNT-100_AM, (iii) Ag/TNT-100_IN, (iv) Ag/TNT-100_NB, (v) Ag/TNT-100_EL (0.1), and (vi) Ag/TNT-100_EL (1), Figure S6: Antibacterial properties of Ag/TNTs with reference to Ag release from the hybrid nanomaterial.
