# Supplementary material for: Influence of Preparation Procedure on Physicochemical and Antibacterial Properties of Titanate Nanotubes Modified with Silver
Source: Nanomaterials (Basel). 2019 May 23;9(5):795. doi: 10.3390/nano9050795 (PMC6566197; doi:10.3390/nano9050795)
Supplement: Supplementary file 1 [file nanomaterials-09-00795-s001.pdf]

nanomaterials

Supplementary Materials

Influence of Preparation Procedure on Physicochemical and Antibacterial Properties of Titanate Nanotubes Modified with Silver

Manu Jose [1], Paulina Sienkiewicz [1], Karolina Szymańska [2], Dominika Darowna [1], Dariusz Moszyński [1], Zofia Lendzion-Bieluń [1], Kacper Szymański [1] and Sylwia Mozia [1,*]

[1] Institute of Inorganic Chemical Technology and Environment Engineering, Faculty of Chemical Technology and Engineering, West Pomeranian University of Technology, Szczecin, ul. Pułaskiego 10, 70-322 Szczecin, Poland; manu.jose@zut.edu.pl (M.J.); paulina.sienkiewicz@zut.edu.pl (P.S.); dominika.darowna@zut.edu.pl (D.D.); dariusz.moszynski@zut.edu.pl (D.M.); zofia.lendzion-bielun@zut.edu.pl (Z.L.-B.); kacper.szymanski@zut.edu.pl (K.S.)

[2] Nanomaterials Physicochemistry Department, Faculty of Chemical Technology and Engineering, West Pomeranian University of Technology, Szczecin, al. Piastów 45, 70-311 Szczecin, Poland; karolina.szymanska@zut.edu.pl

* Correspondence: sylwia.mozia@zut.edu.pl; Tel.: +48-91-449-47-30

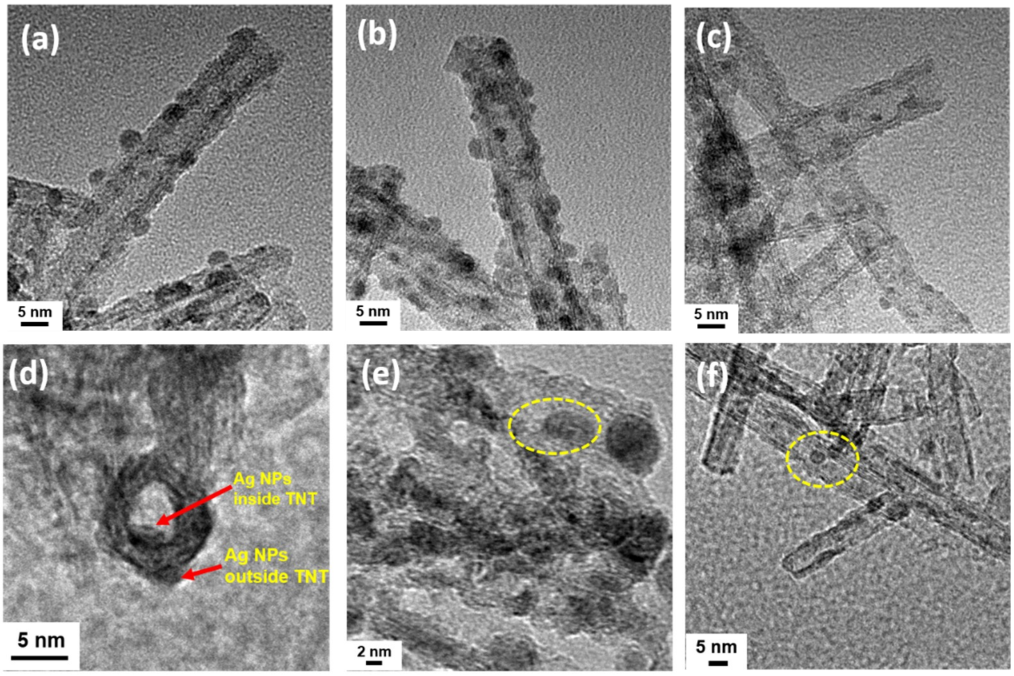

Figure S1. HRTEM image of (a) Ag/TNT-100_IN, (b) Ag/TNT-100_EL(0.1) and (c)-(f) Ag/TNT-100_NB (The circled regions in (e) and (f) represent the Ag NPs anchored to the inner surface of the TNTs. Moreover, in Figure S1(e) the Ag NP blocking the entrance to the TNT can be observed).

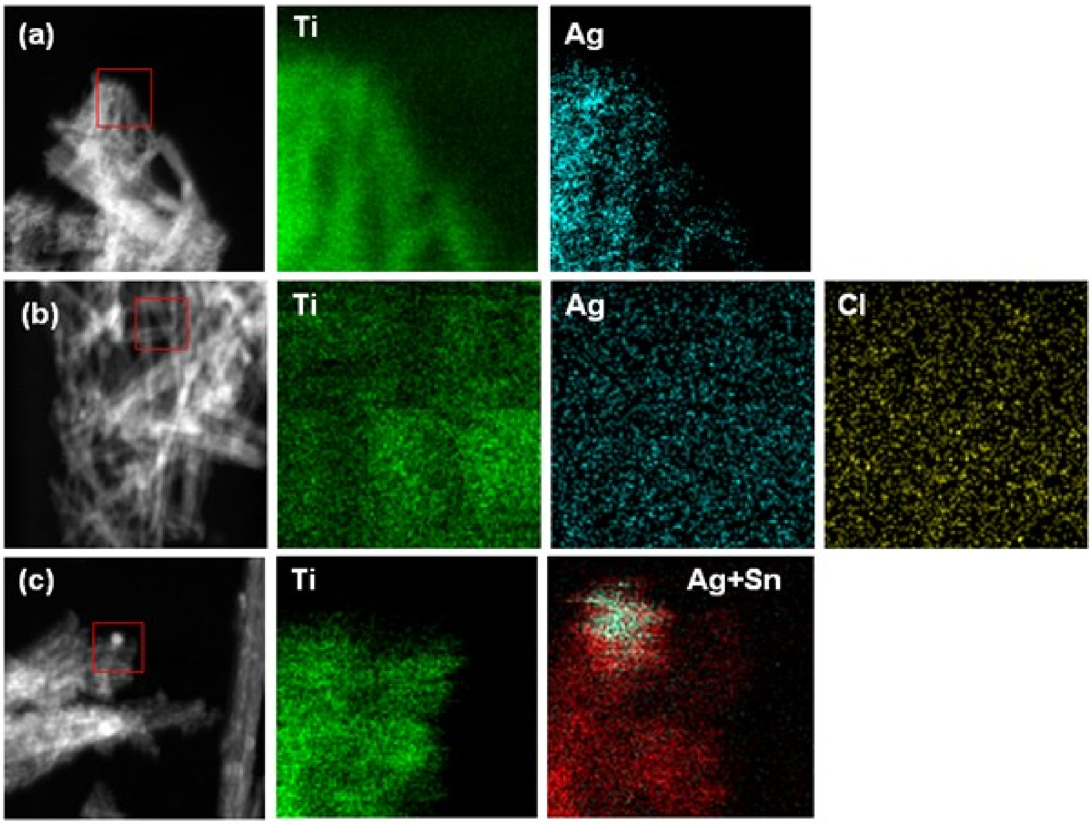

Figure S2. EDS elemental mapping of (a) Ag/TNT-2.5_AM, (b) Ag/TNT-5_SH, (c) Ag/TNT-2.5_EL(1). Scanning transmission electron microscopy (STEM) images with red squares present the scanned area.

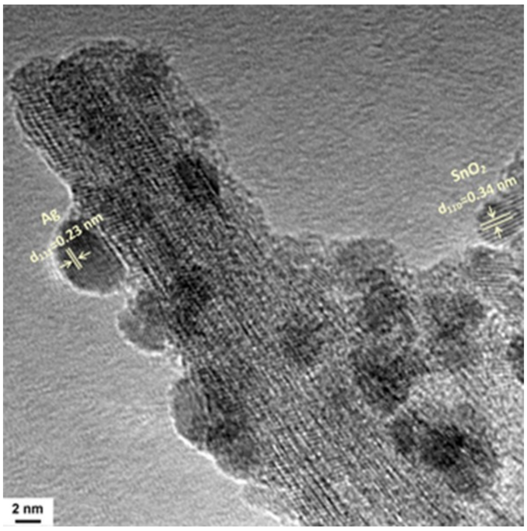

Figure 3. HRTEM image of Ag/TNT-100_EL(1).

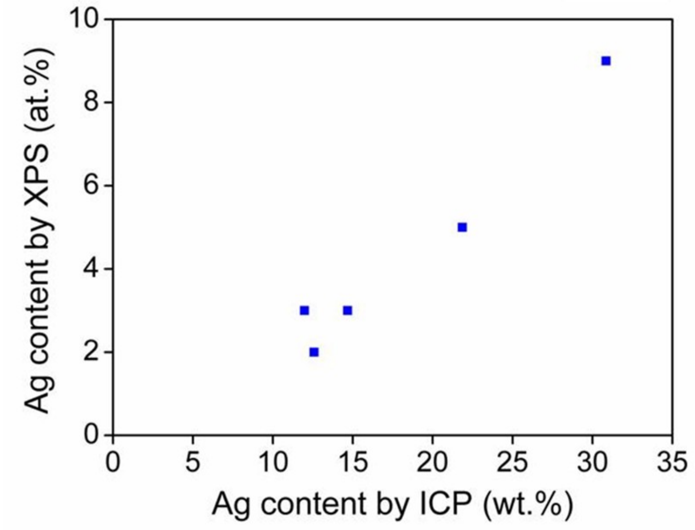

Figure 4. The dependence between Ag content measured by XPS and ICP methods.

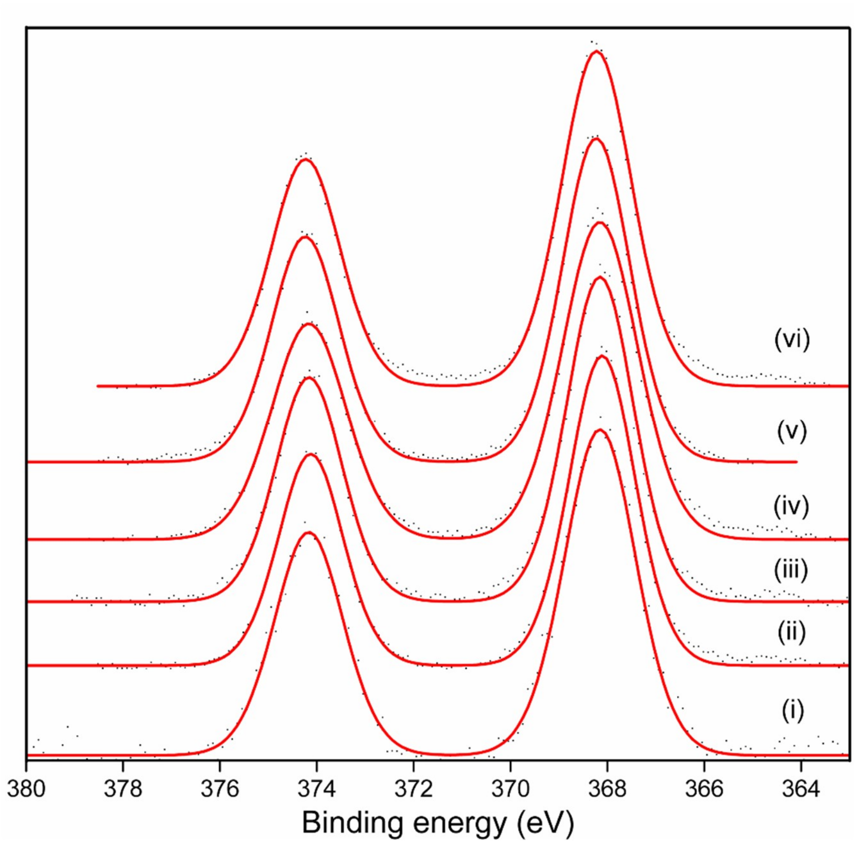

Figure 5. XPS spectra of (i) Ag/TNT-5_SH, (ii) Ag/TNT-100_AM, (iii) Ag/TNT-100_IN, (iv) Ag/TNT-100_NB, (v) Ag/TNT-100_EL(0.1) and (vi) Ag/TNT-100_EL(1).

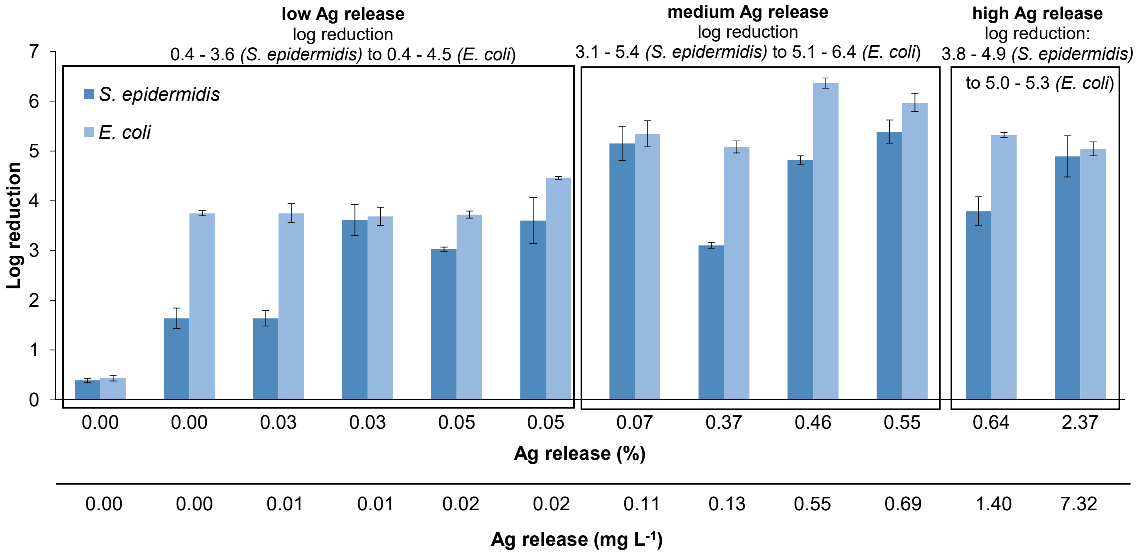

Figure 6. Antibacterial properties of Ag/TNTs with reference to Ag release from the hybrid nanomaterial.
